# Isolation and RNA Extraction of Neurons, Macrophages and Microglia from Larval Zebrafish Brains

**DOI:** 10.3791/57431

**Published:** 2018-04-27

**Authors:** Julie Mazzolini, Kelda Chia, Dirk Sieger

**Affiliations:** ^1^Centre for Discovery Brain Sciences, University of Edinburgh

**Keywords:** Neuroscience, Issue 134, Transcriptome, qPCR, microglia, neurons, RIN, RNA extraction, FACS, zebrafish, development.

## Abstract

To gain a detailed understanding of the role of different CNS cells during development or the establishment and progression of brain pathologies, it is important to isolate these cells without changing their gene expression profile. The zebrafish model provides a large number of transgenic fish lines in which specific cell types are labelled; for example neurons in the NBT:DsRed line or macrophages/microglia in the mpeg1:eGFP line. Furthermore, antibodies have been developed to stain specific cells, such as microglia with the 4C4 antibody.

Here, we describe the isolation of neurons, macrophages and microglia from larval zebrafish brains. Central to this protocol is the avoidance of an enzymatic tissue digestion at 37 °C, which could modify cellular profiles. Instead a mechanical system of tissue homogenization at 4 °C is used. This protocol entails homogenization of brains into cell suspension, their immuno-staining and the isolation of neurons, macrophages and microglia by FACS. Afterwards, we extracted RNA from those cells and evaluated their quality/quantity. We managed to obtain RNA of high quality (RNA Integrity Number (RIN) > 7) to perform qPCR on macrophages/microglia and neurons, and transcriptomic analysis on microglia. This approach enables a better characterization of these cells, as well as a clearer understanding about their role in development and pathologies.

**Figure:**
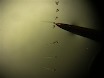


## Introduction

Knowledge on brain development and brain diseases has significantly improved in the last decade since the first quantification of mouse brain transcriptomes^1^. Indeed, genome wide gene expression analysis gives us access to detailed genetic information on brain tissue and cells that can complement and improve observations made with other techniques and tools.

The zebrafish is a potent biological model, easy to breed and to modify genetically; its optical transparency at larval stages allows live imaging observations^2^. Unfortunately, compared to human and mouse, the number of available antibodies to perform immuno-staining is rather low. To remedy this, transgenic zebrafish fish lines are easily made by genetically modifying fish to express fluorescent proteins under cell type specific promoters. Transgenic zebrafish lines have been used in the past to study the role of macrophages and microglia during central nervous system (CNS) development and disease^3^,^4^,^5^,^6^. However, to gain a detailed understanding of these processes we need to understand changes in gene expression in the respective cell types. To this aim, we developed an experimental method to specifically isolate cells like neurons, macrophages and microglia from 3 to 8 days post-fertilization (dpf) larval zebrafish brains. For the establishment of the protocol, we worked with transgenic fish lines that express green fluorescent protein (GFP) in macrophages/microglia under the macrophage-expressed gene promoter (mpeg1:eGFP) and DsRed in neurons under the neural ß-tubulin promoter (NBT:DsRed)^7^,^8^,^9^. Furthermore, we performed immuno-staining of microglia using 4C4, a mouse monoclonal antibody that specifically stains zebrafish microglia^10^,^11^. Afterwards, ribonucleic acid (RNA) is extracted from these cells for further quantitative polymerase chain reaction (qPCR) or transcriptome analyses. This protocol has been designed to efficiently homogenize brain tissue from zebrafish larvae; collect neurons, macrophages/microglia and microglia without alteration of their plasma membrane integrity and finally extract RNA from these cells in high quality (RIN > 7) and quantity to perform genomic analysis. Unlike previously published studies that use trypsin treatment at 37 °C to digest brain tissue^12^,^13^, this protocol promotes work at 4 °C till the RNA extraction step to reduce modifications of the gene expression profile. This step is crucial as microglia and macrophages are highly sensitive cells which respond to changes in their microenvironment immediately by altering their gene expression profile and polarization^14^,^15^,^16^.

The protocol, described here in detail, shows the isolation of neurons, macrophages and microglia from zebrafish larval brains, but virtually, it can be adapted to any other cell present within the brain - either by using transgenic fish lines or labelled with specific antibodies. This method will allow a better characterization of CNS cells through their genome wide gene expression analyses and will help to understand their role during development and brain diseases.

## Protocol

### 1. Sample and Media Preparation

1. Prepare embryo medium (E3) by dissolving 6.4 mM KCl, 0.22 mM NaCl, 0.33 mM CaCl_2_ 2H_2_O, 0.33 mM MgSO_4_ 7H_2_O in H_2_O.
2. Collect zebrafish embryos immediately after fertilization (0 dpf).
  1. Split zebrafish embryos into 50 per 90 mm petri dish. Raise embryos at 28.5 °C in 50 mL of embryo medium (E3) treated with 200 µM 1-phenyl 2-thiourea (PTU), from the end of the first day of development (0 dpf) for the duration of the experiment to inhibit pigmentation.
  2. Change the E3 + PTU medium daily for the duration of the experiment.
  3. Screen mpeg1:eGFP and NBT:DsRed larvae at 2 dpf using a florescent stereomicroscope for the positive transgene expression, GFP^+^ macrophages/microglia and DsRed^+ ^Neurons.
3. Prepare all media the day before the experiment under a tissue culture hood to avoid contamination, then store them at 4 °C.
  1. Prepare media A by dissolving 15 mM Hepes and 25 mM D-Glucose in HBSS 1x.
  2. Prepare density gradient medium (100%) by mixing 9 volumes of the density gradient medium in 1 volume of HBSS 10x.
  3. Prepare the density gradient medium (22%) by diluting 22 mL of density gradient medium (100%) in 88 mL of DPBS 1x.
  4. Prepare DPBS 1x.
  5. Prepare E3 medium + Tricaine by mixing E3 medium with 450 μM Tricaine.

### 2. Homogenization

**Note:** All steps are performed 4 °C.

1. Add 1.5 mL Tricaine (15 mM) to 90 mm Petri dishes containing 50 larvae in 50 mL of E3 embryo medium treated with 200 µM PTU to terminally anesthetize them.
  1. Suck up 10 anesthetized larvae from the petri dishes with a 3 mL Pasteur plastic bulk pipette.
  2. Transfer anesthetized larvae 10 by 10 into a 55-mm petri dish filled with ice-cold E3 embryo medium + Tricaine.
  3. Under a stereomicroscope, align 10 larvae in the center of the petri dish. Then transect larval heads above the yolk-sac using surgical micro-scissors (exclude swim bladder to avoid floating heads).
  4. Suck up heads from the petri dishes with a 3 mL Pasteur plastic bulk pipette. Wait until all heads gather within the pipette tip and then transfer them into a glass homogenizer containing 1 mL ice-cold Media A (transfer in a minimal volume to reduce Media A dilution by E3 + Tricaine). Keep the glass homogenizer on ice. Use one homogenizer per experimental condition.
  5. Replace each small petri dish containing ice cold E3 + Tricaine with a new one every 30 min to assure that transection is performed in cold E3 + Tricaine medium.
  6. Replace the ice-cold Media A in the glass homogenizer when the color starts fading. **Note:** Media A dilution can alter the head tissue due to temperature changes.
  7. Once all heads have been collected ( 600 heads/condition), remove the maximum volume of Media A from the glass homogenizer and replace it with 1 mL of fresh ice-cold Media A.
  8. Disrupt the brain tissue with a tight glass homogenizer on ice. Perform 40 rounds of crushing and turns for 3-5 dpf larvae and 50 for 7 and 8 dpf larvae.
2. Add 2 mL of Media A to cell suspension (1 mL Media A / 200 heads), that will dilute cells and reduce their agglomeration with myelin to facilitate their separation during the centrifugation in density gradient medium.
  1. To eliminate cell agglomeration, run the cell suspension through a 40 µm cell strainer placed on top of a cold 50 mL falcon tube maintained on ice. Repeat this operation 3 times.
  2. Transfer 1 mL of cell suspension into cold 1.5 mL tubes and spin them at 300 g for 10 min at 4 °C.
  3. Remove supernatant using a 10-mL syringe + needle 23G x 1''.
3. Re-suspend the cell pellet with 1 mL of ice-cold 22% density gradient medium gently overlaid by 0.5 mL of ice-cold DPBS 1x (do not mix them, an interphase between both solutions will be seen).
  1. Spin tubes at 950 g without brake and slow acceleration for 30 min at 4 °C. **Note:** This step separates myelin from other cells by sequestering it at the interphase of DPBS 1x and 22% density gradient medium, whereas cells will pellet at the bottom of the tube. Myelin removal is more efficient when cell concentration is not too high.
  2. Using a 10-mL syringe + needle 23G x 1'' discard the maximum of DPBS, density gradient medium and myelin trapped at their interphase.
  3. Wash cells with 0.5 mL of Media A + 2% of normal goat serum (NGS), then spin tubes at 300 g for 10 min at 4 °C.
  4. Discard the maximum of the supernatant, then pool all cell pellets from the same experimental condition together in 1 mL of Media A + 2% NGS.
4. If the cells of interest express a fluorescent protein like macrophages/microglia from mpeg1:eGFP or neurons from NBT:DsRed transgenic fish (screening of transgenic fish see 1.1.3), run the cell suspension through a 35 µm cell strainer cap and transfer them into a cold 5 mL FACS tubes on ice, protected from light. **Note:** Alternatively, immuno-staining of microglia can be performed.

### 3. Microglia Immuno-staining

**Note:** All steps are performed at 4 °C.

1. Re-suspend the cell pellet with 0.3 mL of Media A + 2% NGS. Split them in 3 x 1.5 mL tubes: one for unstained cells to measure auto-fluorescence from cells of interest, second for the secondary antibody (1/200) to measure the non-specific binding of the secondary antibody to microglia and third as a test (4C4 mouse monoclonal antibody (microglia specific) (1/20) + secondary antibody (1/200)).
  1. Add Low Endotoxin, Azide-Free (LEAF) at 1% to cells (all tubes) to block CD16/CD32 interactions with the Fc domain of immunoglobulins. Incubate cells for 10 min with gentle agitation every 5 min.
  2. Add the 4C4 antibody (1/20) to cells (tube 3) and incubate for 30 min with gentle agitation every 10 min.
  3. Spin tubes at 300 g for 10 min at 4 °C, then discard the supernatant.
  4. Wash once with 0.5 mL of Media A + 2% NGS, then spin tubes at 300 g for 10 min at 4 °C.
  5. Re-suspend cell pellet with 0.5 ml of Media A + 2% NGS and incubate cells with LEAF at 1% for 10 min with gentle agitation every 5 min.
  6. Add secondary antibody (1/200) to cells (tube 2 and 3). Incubate cells for 30 min with gentle agitation every 10 min and light protection.
  7. Spin tubes at 300 g for 10 min at 4 °C, then discard supernatant.
  8. Wash twice with 0.5 mL of Media A + 2% NGS, then re-suspend cell pellet with 1 mL of Media A + 2% NGS.
2. Run cell suspension through a 35 µm cell strainer cap and transfer them into cold 5 mL FACS tubes on ice, protected from light.

### 4. Cell Sorting (FACS)

**Note:** Perform all steps at 4 °C.

1. Sort neurons, macrophages/microglia and microglia using a FACS. **Note:** This step is usually performed by a staff member of the FACS facility and settings depend on the type of equipment used.
  1. Add DAPI at a concentration of 1 µg/mL in each FACS tube to label dead cells.
  2. Set up FACS and sort neurons, macrophages/microglia or microglia from all brain cells. Separate cells from debris in function of their size and granularity, then gate single-cells by forward scatter and side scatter. Exclude dead cells by DAPI labelling from live cells. Identify neurons, macrophages/microglia or microglia by their respective positive staining.
2. Collect cells in 1.5 mL tubes containing 1 mL of ice-cold Media A + 2% NGS on ice. Use different tubes for each cell type.
  1. Spin tubes at 300 g for 10 min at 4 °C and then discard the supernatant.
  2. Wash once with 0.5 mL of Media A then discard the maximum of supernatant.

### 5. RNA Extraction

1. Extract RNA from the different cell types separated by FACS. For RNA extraction use a specific kit and follow the manufacturer's guidelines. Ensure to work in an RNase free environment by cleaning workspace and pipettes with a RNase decontamination product and use filter tips.
  1. Freshly prepare 1 mL of lysis buffer supplemented with 50 µM β-mercaptoethanol. **Note:** Here, β-mercaptoethanol is added to reduce RNA degradation.
  2. Prepare 70% and 80% ethanol solution using RNase free water.
  3. Lyse cells with 75 µL of lysis buffer supplemented with 50 µM β-mercaptoethanol. Then use a shredder to improve cell disruption.
  4. Continue RNA extraction according to manufacturer's manual.
  5. At the end of the protocol, elute the RNA with 14 µL of RNase free water to obtain a sufficient RNA concentration.

## Representative Results

The described protocol is a straightforward approach to isolate neurons, macrophages and microglia from zebrafish larval brains. From these isolated cells, significant amounts of high quality (RIN > 7) RNA were extracted. The aim of this protocol is to isolate different types of cells from the CNS, with minimal modification of their gene expression profile to analyze and characterize cell properties and functions. Therefore, the entire protocol is performed at 4 °C with a mechanical brain tissue homogenization. This method has been successfully used for two studies performed in the laboratory. In the first study, neurons and macrophages/microglia were isolated from 8 dpf mpeg1:eGFP^+^/NBT:DsRed^+^ larvae (Figure 1). FACS allowed cell separation from debris in function of their size (FSC-A) and granularity (SSC-A) (Figure 1**A**). Single cells were then separated from doublets or cell agglomerates (Figure 1**B**). From the single cell population, a gate was drawn to eliminate dead cells (DAPI^+^). The corresponding dot plot revealed that this experimental protocol preserves cell plasma membrane integrity, as the rate of dead cells is only 26.7% (Figure 1**C**). Finally, neurons (DsRed^+^) and macrophages/microglia (GFP^+^) were easily segregated from the live cell population gates. The neuron population (23.1 %) appeared to be more prominent than the macrophages/microglia population (1.56 %) within the brain (Figure 1**D**). This protocol has allowed to isolate RNA from those cells to perform subsequent qPCR analyses to compare the expression of specific genes between neurons and macrophages/microglia. Figure 2 shows neuronal and macrophages/microglia gene expression levels of *proliferating cell nuclear antigen (pcna)*against the *β-actin* house-keeping gene as an example.

For the second study this method focused on microglia isolation from 3, 5 and 7 dpf larval brains. In contrast to the experiment described above, cells were isolated by immuno-staining using 4C4, an antibody which specifically labels microglia (Figure 3 **A-D**). As previously described, microglia (4C4^+^) were selected from live cells and collected (Figure 3**D**). Microglia numbers within zebrafish larval brains are variable (**Table 1**), and very low at 3 dpf (∼ 25 per fish). Quality and quantity of extracted RNA from those cells were measured using a micro-capillary electrophoresis based system. Results obtained of extracted RNA from microglia of 5 dpf larval zebrafish brains have been provided to illustrate an example of RNA analysis (**Table 1** (5 dpf; experiment 4)). Figure 4 shows the electrophoresis trace and its graphic representation obtained for this sample with a clear visualization of ribosomal RNA (28s and 18s). This data is necessary to calculate sample RIN and to determine RNA concentration. **Table 1** summarizes the number of isolated microglia per fish, the amount of RNA per microglia and the RIN score obtained for each different experiment at 3, 5 and 7 dpf. The amount and the quality of extracted RNA from isolated microglia using this method allowed us to amplify the RNA into cDNA using a kit. Quality and quantity tests provided by Edinburgh Genomics confirm that the amplified cDNA is of sufficient quality for library preparation and subsequent sequencing. Figure 5 shows the size distribution of cDNA fragments and their amount measured using an electrophoretic system. In this sample, the cDNA had a mean size of 299bp at a concentration of 36100 pmol/l. **Table 2** illustrates respectively quality and quantity tests made on amplified cDNA from RNA samples (**Table 1** (5 dpf; experiment 4)). The amplified cDNA has been used successfully for sequencing.

Several studies performed in the laboratory confirmed that the quality and quantity of extracted RNA from neurons, macrophages and microglia can be used for subsequent qPCR and genome wide gene expression analyses. Therefore, this experimental protocol can be used to reliably isolate different types of CNS cells without altering their membrane integrity and limiting modification of their gene expression profile.

**Figure:**
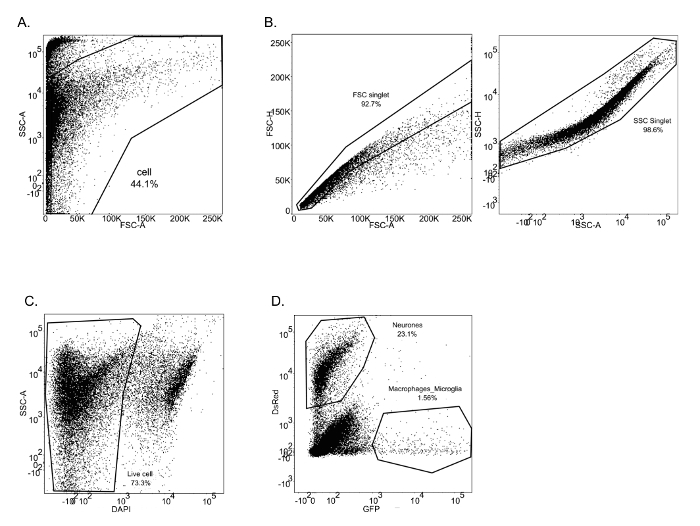

Figure 1**: FACS sorting for neurons and macrophages/microglia from mpeg1:GFP^+^/NBT:DsRed^+^ 8 dpf zebrafish larvae.** (A-C) Successive gating shows sequential selection of all brain cells (A), single cells by forward scatter and side scatter (B). (C) Dead cells were excluded by DAPI labelling. (D) Neurons and macrophages/microglia were identified respectively by DsRed and GFP positive staining. Please click here to view a larger version of this figure.

**Figure:**
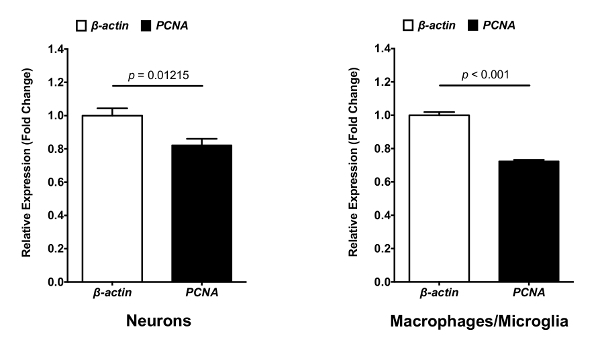

Figure 2**: Gene expression analysis for *pcna* and *β-actin* in neurons and macrophages/microglia.** RNA from isolated neurons and macrophages/microglia can be transcribed into cDNA for use in quantitative PCR analysis. mRNA expression levels of *pcna* against *β-actin* house-keeping gene in isolated neurons and macrophages/microglia determined by qPCR (N = 3). Fold change was measured using the comparative (ΔΔCT) method. Error bar represent mean ± SEM. Please click here to view a larger version of this figure.

**Figure:**
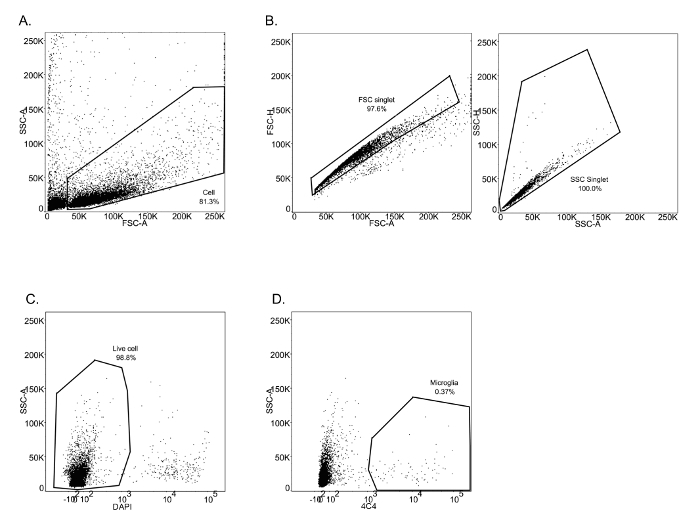

Figure 3**: FACS sorting for microglia from 3 dpf zebrafish larvae.** (A-C) Successive gating show sequential selection of all brain cells (A), single cells by forward scatter and side scatter (B). (C) Dead cells were excluded by DAPI labelling. (D) Microglia were identified by 4C4 positive staining. Please click here to view a larger version of this figure.

**Figure:**
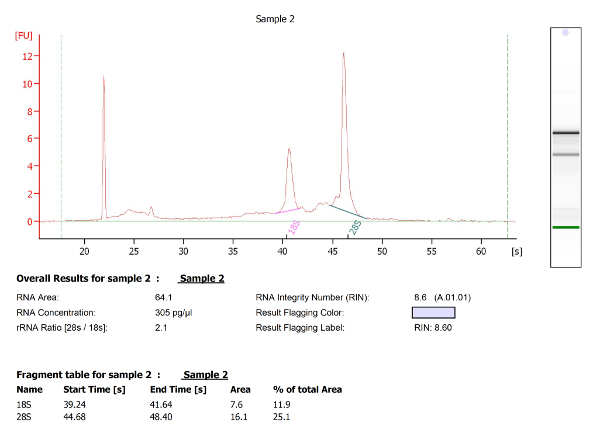

Figure 4**: Micro-capillary electrophoresis results of extracted RNA from 5 dpf zebrafish microglia. **The two tall peaks are the 18S and 28S ribosomal RNA. RNA integrity number (RIN) was automatically calculated by the bioanalyzer software using the generated ratio of the 18S and 28S ribosomal subunits and the analysis of the entire electrophoretic trace. Microglia RNA has a RIN 8.6. Please click here to view a larger version of this figure.

**Figure:**
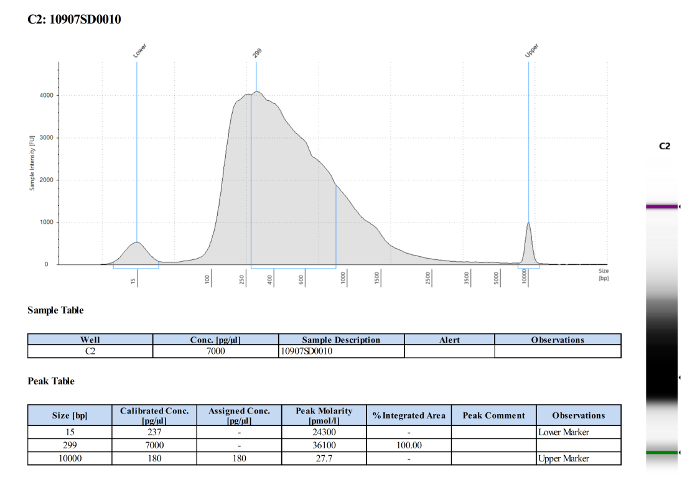

Figure 5**: Quality and quantity tests of amplified cDNA from RNA sample of 5 dpf zebrafish microglia.** The image shows the cDNA fragment size distribution of the analyzed sample with a mean size of 299 bp. Please click here to view a larger version of this figure.

**Table:** 

**Condition**	**Experiment**	**Fish number**	**Cell number**	**Cell number per fish**	**RNA concentration (pg/ul)**	**Total RNA (pg)**	**RNA amount per cell (pg)**	**RIN score**
3dpf	1	700	11922	17.03	126	1512	0.13	8
3dpf	2	600	22527	37.55	253	3036	0.13	7.9
3dpf	3	600	18688	31.15	255	3060	0.16	7.7
3dpf	4	600	11121	18.54	189	2268	0.20	7.8
3dpf	5	600	15581	25.97	131	1572	0.10	8.4
3dpf	6	600	11965	19.94	256	3072	0.26	8.2
5dpf	1	600	58629	97.72	362	4344	0.07	7.4
5dpf	2	600	32510	54.18	348	4176	0.13	8.1
5dpf	3	600	77884	129.81	594	7128	0.09	8.3
5dpf	4	600	50755	84.59	305	3660	0.07	8.6
5dpf	5	600	44967	74.95	134	1608	0.04	7.6
5dpf	6	600	51031	85.05	163	1956	0.04	7.9
7dpf	1	600	60496	100.83	183	2196	0.04	7.6
7dpf	2	450	55517	123.37	183	2196	0.04	7.8
7dpf	3	600	88897	148.16	465	5580	0.06	8.1
7dpf	4	600	63008	105.01	356	4272	0.07	8.4
7dpf	5	350	34956	99.87	245	2940	0.08	8.1
7dpf	6	600	63887	106.48	341	4092	0.06	7.8

**Table 1: Summary of microglia isolation and RNA extraction data from 3, 5 and 7 dpf zebrafish larvae.**

**Table:** 

**Internal Sample ID**	**External Sample ID**	**Qubit (ng/ul)**	**Qubit(ng/ul)**	**Average concentration (ng/ul)**	**Volume (ul)**	**ug received**	**Pass/fail for minimum concentration**	**Pass/fail for minimim quantity**	**Pass/fail for recommended quantity**
10907SD0010	5 dpf (experiment 4)	58.8	58.4	58.6	30	1.76	Pass	Pass	Pass
**Sample Requirements for Library Prep:**
**Library Prep**	**Minimum Quantity (ng)**	**Recommended Quantity (ng)**	**Minimum Concentration ng/uL**						
TruSeq Nano gel free 350 bp insert library from cDNA	600	1100	10						

**Table 2: Quantity tests of amplified cDNA from RNA sample of 5 dpf zebrafish microglia.**

## Discussion

The experimental protocol described here represents a robust and efficient method to isolate brain cells from zebrafish larvae from 3 to 8 dpf. Importantly, this is the first protocol that allows the specific isolation of microglia from larval zebrafish brains. The protocol is designed to preserve cell membrane integrity and to minimize potential modifications of gene expression occurring during the processing. This last point is crucial for the relevance of results based on the analysis of those isolated cellular genomic profiles. Indeed, microglia and macrophage polarization are strongly influenced by their microenvironment. At 37 °C, these cells would have changed their gene expression profile in response to experimental conditions (injury (transection) response). Therefore, it was crucial to perform this experiment at 4 °C prior to RNA extraction, to slow down cellular processes and metabolic activities. Furthermore, the mechanical brain tissue homogenization at 4 °C was chosen instead of enzymatic tissue digestion at 37 °C to avoid any impact on gene expression profiles.

It is important to highlight that this method is very quick; within a day several brain cell populations can be isolated from at least two different experimental conditions and their RNA extracted. The total length of the protocol depends on the number of larvae used for each condition as the transection of larval heads is the limiting step (∼ 350 heads/h). In general, to work with microglia from 3, 5 and 7 dpf it is recommended to transect ∼ 600 heads per test to get enough RNA to extract (**Table 1**). As microglia represent the cell type with the lowest yield (approx. 112 cells per head at 7 dpf), the number of heads can be reduced for other cell types including macrophages (approx. 170 cells per head at 7 dpf).

Another advantage of this protocol is that once settings on the FACS sorter have been established, the same settings can be used for different experiments. It has been observed that cell populations fit perfectly from one experiment to another with gates previously designed, showing the reproducibility of experiments using this method.

A slight disadvantage of this method is the relatively low amount of RNA that is harvested. However, this limitation is more a biological issue than a technical issue, as the number of microglia is very low at early stages of brain development (**Table 1**). Because of this low quantity of RNA collected, amplification steps need to be considered to perform genome wide gene expression analysis. Fortunately, these amplification steps produce sufficient amounts of high quality cDNA. Thus, global changes in the gene expression profiles of isolated cells can be studied.

In conclusion, this protocol provides a robust method to isolate and study various CNS cell types from larval zebrafish brains. This can be applied to gain a deeper understanding of these cells during development as well as to study their role in disease.

## Disclosures

The authors have nothing to disclose.
